# Sex-differences in the management and clinical outcome among patients with acute coronary syndrome

**DOI:** 10.1186/s12872-021-02433-4

**Published:** 2021-12-20

**Authors:** Yunliang Zou, Wenjian Zhu, Jing Zeng, Junyu Lin, Siping Dai

**Affiliations:** 1 Department of Emergency, The Third People’s Hospital of Huizhou, The Affiliated Hospital of Guangzhou Medical University, Huizhou, Guangdong China; 2 Department of Cardiology, The Third People’s Hospital of Huizhou, The Affiliated Hospital of Guangzhou Medical University, Huizhou, Guangdong China

**Keywords:** Acute coronary syndrome, Sex, Clinical outcome, Mortality, Heart failure

## Abstract

**Background:**

The current study was to compare the management and clinical outcome between women and men with acute coronary syndrome (ACS).

**Method:**

This was a retrospective study. Patients with ACS presented to the emergency department were enrolled. Management and clinical outcomes (including mortality and acute decompensated heart failure [ADHF]) were compared between women and men.

**Results:**

A total of 686 patients were included and women accounted for 38.5% (n = 264). Women were less likely to receive ticagrelor at the emergency department (18.2% vs 25.1%). Duration from arrival at the emergency department to undergo electrocardiogram was longer in women (7.5 min vs 5.3 min). The duration from symptom onset to undergo percutaneous coronary intervention was longer in women (14.4 h vs 7.2 h). After adjusting for covariates, odds ratio (OR) for cardiovascular mortality was 0.42 (95% confidence interval [CI] 0.37–1.02) and ADHF was 0.63 (95% CI 0.55–1.01) for women vs men. Socioeconomic status, duration from symptom onset to arrive at the emergency department, and management at the emergency department were the important factors contributing to the sex-differences in clinical outcome.

**Conclusion:**

Among ACS patients undergoing PCI, there was no sex-difference in in-hospital clinical outcome after adjusting for covariates. Future studies are needed to evaluate whether improving management at the emergency department can improve clinical outcomes in women and men with ACS.

## Background

Despite advancement has been achieved in the last decade, acute coronary syndrome (ACS) remains a leading cause of morbidity and mortality worldwide [1–5]. The standardized incidence of ACS in Chinese adults was approximately 2185/million [6], and the mortality rate of ACS was approximately 6% [3], which was comparable to the developed countries [4, 5]. One recent report from the CCC-ACS project (Improving Care for Cardiovascular Disease in China-Acute Coronary Syndrome) indicated that compared to men, women with ACS were less likely to receive guideline-recommended therapy, which attributed to a higher mortality rate during hospitalization in women [7]. While this study did not account for the severity of coronary artery stenosis [7]. Indeed, results from prior study suggested that after adjusting for severity of coronary artery stenosis, there was no difference in 30-day mortality between women and men [8].

Sex-differences in ACS have been extensively evaluated [7, 9–14]. Nonetheless, most of prior studies were from western populations and data on Chinese ACS populations are remarkably limited. In addition, whether there are sex-differences in ACS management at the emergency department are unknown. Notably, effective management at the emergency department is essential to improve clinical outcomes for ACS patients [15, 16]. Considering the increasing prevalence of ACS in China and differences in clinical outcome between women and men [2, 3], it is clinically important to investigate whether there were sex-differences in management at the emergency department, and whether these differences would influence clinical outcome among ACS patients.

Herein, we performed a retrospective study to include ACS patients presented to the emergency department of our hospital. The management at the emergency and inpatient department and the clinical outcomes during hospitalization were evaluated in women and men.

## Methods

### Study participants

**Fig. 1:**
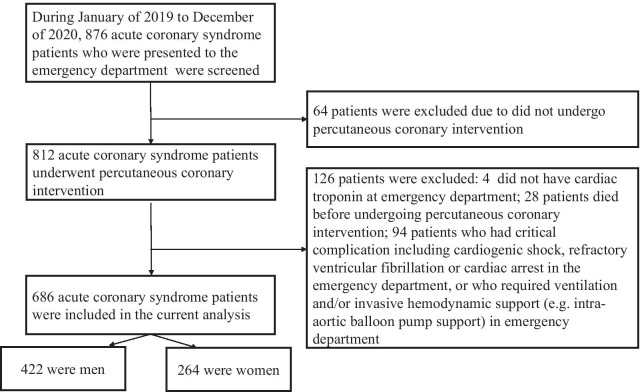
Study flowchart

The current study was approved by the Institutional Review Board (IRB; No. 20190768A02) of the Third People’s Hospital of Huizhou. Since this was a retrospective study, the written consent form was waived by the IRB. All the experiment was conducted according to the Helsinki Declaration. Patients with ACS who were presented to the emergency department during January of 2019 to December of 2020 were screened. The included criteria were as follow: > 18 years old and underwent percutaneous coronary intervention (PCI) during hospitalization. The excluded criteria were as follow: patients who did not have cardiac troponin measurement at the emergency department; patients who died before undergoing PCI; patients who had critical complication including cardiogenic shock, refractory ventricular fibrillation or cardiac arrest at the emergency department, or who required ventilation and/or invasive hemodynamic support (e.g. intra-aortic balloon pump support) at the emergency department (Fig. 1).

### Data collection

Data were extracted from the electronic health record of the emergency department and the inpatient healthcare system by two study investigators. Baseline data, including demographics, vital sign at the emergency department, socioeconomic status (education degree and insurance status), duration since symptom onset to the arrival of emergency department, approach to emergency department (emergency medical service, EMS), management at the emergency department, comorbid conditions (smoking, hypertension, dyslipidemia, diabetes mellitus [DM], atrial fibrillation, chronic kidney disease [CKD], chronic obstructive pulmonary disease [COPD], coronary heart disease [CHD], ischemic stroke, peripheral arterial disease, heart failure, and prior PCI), were collected. Laboratory included high sensitive cardiac troponin-T (Hs-cTnT) and N-terminal pro-B type natriuretic peptide (NT-proBNP) at the emergency and inpatient department, glycated hemoglobin A1c (HbA1c), lipid panel, serum levels of blood urine nitrogen, creatinine, and C-reactive protein (CRP). Creatinine was used to calculate estimated glomerular filtration rate (eGFR) using the Modification of Diet in Renal Disease formula. Procedural characteristics included duration since symptom onset to undergo PCI, arterial access site, number of coronary arteries ≥ 70% stenosis, lesion location and lesion length, type and number of stent implanted, TIMI flow grade of pre- and post-PCI, and antiplatelet loading at peri-PCI period. Medications used at the emergency and inpatient department were extracted from healthcare system.

### Clinical outcome

Clinical outcome of current study included cardiovascular and non-cardiovascular mortality and acute decompensated heart failure (ADHF) during hospitalization. All the clinical outcomes were adjudicated by an independent cardiologist who did not participate in the current study.

### Statistical analysis

Continuous variable with normal distribution was presented as mean ± standard deviation otherwise was presented as median (interquartile range). Categorical variables were presented as number and percentage. Participants were divided by sex and between-group differences were evaluated by Student t test or Mann–Whitney U text for continuous variables, and chi-squared test for categorial variables. Multivariable regression analysis was performed to evaluate the relationship between baseline characteristics and sex. Men was served as the reference group. Odds ratio (OR) and 95% confidence interval (CI) was reported. The incidence rate of clinical outcome was compared by sex. To examine the factors associated with the sex-difference in clinical outcome, sequential cumulative adjustment was performed as previously described [17]. Specifically, in the model 1, age was adjusted; in the model 2, age plus socioeconomic status was adjusted; in the model 3, factors in the model 1 and 2 and duration since symptom onset to emergency department and the approach to the emergency department was adjusted; in the model 4, factors in prior 3 models plus management at the emergency department was adjusted; in the model 5, factors in prior 4 models plus medical management in the inpatient department was adjusted; in the model 6, factors in prior 5 models plus procedural characteristics was adjusted. Odds ratio and 95% CI was reported. All the analyses were conducted using the SPSS 23.0 statistical software and a two-sided *P* value < 0.05 was considered as statistical significance.

## Results

### Comparisons of baseline characteristics by sex

A total of 686 patients were included and women accounted for 38.5% (n = 264; Fig. 1). Compared to men (Table 1), women were older, less likely to graduate from college and to have health insurance. In addition, they were more likely to present to the emergency department at a longer duration since symptom onset and less likely to use EMS to the emergency department. Women were less likely to smoke and have COPD and CHD, and they had a higher serum level of Hs-cTNT, NT-proBNP and CRP.

**Table 1:** Baseline characteristics comparisons

Variables	Men (n = 422)	Women (n = 264)	*P*-value
Age (years)	57.3 ± 12.4	60.9 ± 13.8	0.03
Education ≥ College, n (%)	124 (29.4)	62 (23.5)	0.01
Health insurance, n (%)	387 (91.7)	226 (85.6)	0.02
Duration since symptom onset to emergency department (h)*	2.7 (0.9–5.2)	3.6 (1.3–6.0)	0.009
Emergency medical service, n (%)	166 (39.6)	82 (31.1)	0.04
Systolic blood pressure (mm Hg)	135 ± 14	137 ± 15	0.51
Diastolic blood pressure (mm Hg)	79 ± 17	77 ± 16	0.38
Heart rate (beat per minute)	97 ± 20	99 ± 19	0.17
Smoking, n (%)	228 (54.0)	15 (5.7)	< 0.001
Hypertension, n (%)	251 (59.5)	165 (62.5)	0.62
Dyslipidemia, n (%)	157 (37.2)	95 (36.0)	0.93
Diabetes mellitus, n (%)	124 (29.4)	81 (30.7)	0.86
Atrial fibrillation, n (%)	40 (9.5)	31 (11.7)	0.70
COPD, n (%)	69 (16.4)	5 (1.9)	0.006
Chronic kidney disease, n (%)	78 (18.5)	58 (22.0)	0.09
Coronary heart disease, n (%)	102 (24.2)	43 (16.3)	0.02
Ischemic stroke, n (%)	60 (14.2)	35 (13.3)	0.49
PAD, n (%)	52 (12.3)	38 (14.4)	0.18
Heart failure, n (%)	29 (6.9)	19 (7.2)	0.74
Prior PCI, n (%)	48 (11.4)	25 (9.5)	0.37
Hs-cTNT at ED (pg/mL)*	59 (33–267)	76 (44–304)	0.003
NT-proBNP at ED (pg/mL)*	408 (178–836)	521 (233–962)	0.009
Hs-cTNT inpatient (pg/mL)*	86 (57–367)	112 (83–489)	< 0.001
NT-proBNP inpatient (pg/mL)*	535 (260–1129)	734 (495–1362)	< 0.001
HbA1c (%)	5.9 ± 0.6	6.0 ± 0.6	0.81
Total cholesterol (mmol/L)	5.0 ± 0.9	5.1 ± 1.0	0.36
LDL-C (mmol/L)	3.1 ± 0.5	3.1 ± 0.6	0.44
HDL-C (mmol/L)	1.1 ± 0.4	1.0 ± 0.4	0.70
Triglyceride (mmol/L)*	1.9 (0.7–2.8)	1.9 (0.6–2.9)	0.15
C-reactive protein (mg/dL)*	7.6 (3.5–20.4)	12.3 (6.4–31.8)	< 0.001
Blood urine nitrogen (mg/dL)	7.1 ± 2.3	8.0 ± 2.6	0.82
Creatinine (umol/L)	80.4 ± 16.3	79.6 ± 17.9	0.35
eGFR (ml/min/1.73m^2^)	68.3 ± 14.5	64.2 ± 12.7	0.06

COPD, chronic obstructive pulmonary disease; PAD, peripheral arterial disease; PCI, percutaneous coronary intervention; Hs-cTnT, high sensitivity cardiac troponin-T; NT-proBNP, N-terminal pro B-type natriuretic peptide; ED, emergency department; HbA1c, glycated hemoglobin A1c; LDL-C, low-density lipoprotein cholesterol; HDL-C, high density lipoprotein cholesterol; eGFR, estimated glomerular filtration rate

*Presented as median (interquartile range)

### Comparisons of management by sex

Compared to men (Table 2), women were less likely to receive ticagrelor loading at the emergency department. The duration since arrival at emergency department to first electrocardiogram and Hs-cTNT test were longer in women, as was to receive cardiologist consultation. There were no differences in in-hospital medication use between women and men.

**Table 2:** Comparisons of management in emergency and inpatient department by sex

Variables	Men (n = 422)	Women (n = 264)	*P*-value
*Emergency department*			
Aspirin loading, n (%)	326 (77.3)	191 (72.3)	0.18
Clopidogrel loading, n (%)	187 (44.3)	127 (48.1)	0.69
Ticagrelor loading, n (%)	106 (25.1)	48 (18.2)	0.03
Nitroglycerin IV, n (%)	126 (29.9)	72 (27.3)	0.24
Morphine IV, n (%)	30 (7.1)	25 (9.5)	0.11
Duration since arrived at ED to first ECG test (min)*	5.7 (3.1–10.2)	7.5 (4.7–12.5)	0.006
Duration since arrival at ED to first Hs-cTNT test (min)*	7.0 (4.9–13.5)	9.1 (5.6–15.3)	0.01
Duration since arrival at ED to Cardiologist consultation (min)*	15.3 (8.5–19.1)	17.8 (9.6–20.7)	0.03
*Inpatient department*			
Aspirin, n (%)	422 (100)	264 (100)	0.99
Clopidogrel, n (%)	367 (87.0)	225 (85.2)	0.86
Ticagrelor, n (%)	50 (11.8)	25 (9.5)	0.59
Statins, n (%)	412 (97.6)	253 (95.8)	0.72
Betablocker, n (%)	336 (79.6)	205 (77.7)	0.48
RASi, n (%)	252 (59.7)	138 (52.3)	0.08
PPI, n (%)	129 (30.6)	79 (29.9)	0.70
Antidiabetic, n (%)	113 (26.8)	73 (27.7)	0.83
Anticoagulant, n (%)	25 (5.9)	17 (6.4)	0.64
Oxygen supplement, n (%)	73 (17.3)	58 (22.0)	0.06

IV, intravenous; ED, emergency department; ECG, electrocardiogram; Hs-cTNT, high sensitivity cardiac troponin-T; RASi, renin-angiotensin-system inhibitor; PPI, proton pump inhibitor

*Presented as median (interquartile range)

### Comparisons of procedural characteristics by sex

The proportion of patients presented with ST-segment elevation myocardial infarction (STEMI), non-STEMI and unstable angina were comparable by sex (Table 3). The duration since symptom onset to undergo PCI was longer in women with STEMI. Women were less likely to use radial artery access. There were no sex-differences in antiplatelet loading at peri-PCI period, post-PCI TIMI flow grade, and the number and percentage of drug-eluting stent used.

**Table 3:** Comparisons of procedural characteristics by sex

Variables	Men (n = 422)	Women (n = 264)	*P*-value
ST-segment elevation MI, n (%)	172 (40.8)	111 (42.0)	0.46
Non-ST-segment elevation MI, n (%)	156 (37.0)	94 (35.6)	0.60
Unstable angina, n (%)	94 (22.2)	59 (22.3)	0.89
Duration since symptom onset to undergo PCI (hour)*	7.2 (4.8–55.2)	14.4 (9.6–60)	0.04
Radial artery access, n (%)	395 (93.6)	223 (84.5)	0.03
Number of coronary arteries ≥ 70% stenosis	1.7 ± 0.6	1.8 ± 0.6	0.58
Lesion length, mm	25.6 ± 6.1	24.8 ± 5.8	0.31
Lesion locations			
Left main, n (%)	83 (19.7)	56 (21.2)	0.62
LAD, n (%)	186 (44.1)	120 (45.5)	0.44
LCX, n (%)	178 (42.2)	115 (43.6)	0.39
RCA, n (%)	209 (49.5)	138 (52.3)	0.08
Pre-PCI TIMI flow			
Grade 3	0	0	–
Grade 2	59 (14.0)	43 (16.3)	0.26
Grade 1	203 (48.1)	123 (46.6)	0.15
Grade 0	160 (37.9)	98 (37.1)	0.93
Post-PCI TIMI flow			
Grade 3	403 (95.5)	243 (92.0)	0.07
Grade 2	19 (4.5)	21 (8.0)	0.36
Grade 1	0	0	–
Grade 0	0	0	–
Oral antiplatelet loading at peri-PCI period, n (%)	74 (17.5)	43 (16.3)	0.68
Glycoprotein IIb/IIIa inhibitor, n (%)	28 (6.6)	19 (7.2)	0.23
Number of stents implanted	1.8 ± 0.7	1.9 ± 0.7	0.91
Drug-eluting stent, n (%)	418 (99.1)	260 (98.5)	0.83

PCI, percutaneous coronary intervention; MI, myocardial infarction; LAD, left anterior descending; LCX, left circumflex; RCA, right coronary artery

*Presented as median (interquartile range) for STEMI patients only

### Associations between baseline characteristics and sex

**Fig. 2:**
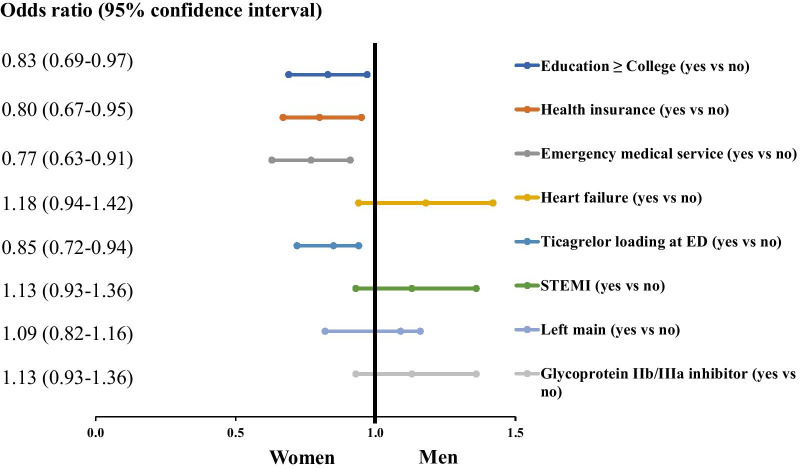
Association of baseline characteristics and sex. STEMI, ST-segment elevation myocardial infarction

After multivariable regression analyses (Fig. 2), female sex was associated with lower odds of college graduation, having health insurance, using EMS, and receiving ticagrelor loading at the emergency department.

### Comparison of clinical outcome by sex

Compared to men (Table 4), women had a higher incidence rate of cardiovascular mortality and ADHF during hospitalization, which together contributed to a higher composite clinical outcome. In the unadjusted model, the crude odds ratio for cardiovascular mortality was 2.57 (95% CI 1.09–6.27), ADHF was 1.65 (95% CI 1.02–2.66) and composite clinical outcome was 1.91 (95% CI 1.25–2.92), respectively.

**Table 4:** Comparisons of clinical outcome by sex

Clinical outcome	Men (n = 422)	Women (n = 264)	OR (95% CI)	*P*-value
CV mortality, n (%)	9 (2.1)	14 (5.3)	2.57 (1.09–6.27)	0.03
Non-CV mortality, n (%)	2 (0.5)	2 (0.8)	1.60 (0.17–15.5)	0.66
ADHF, n (%)	39 (9.2)	38 (14.4)	1.65 (1.02–2.66)	0.04
Composite, n (%)	50 (11.8)	54 (20.5)	1.91 (1.25–2.92)	0.003

CV, cardiovascular; ADHF, acute decompensated heart failure; OR, odds ratio; CI, confidence interval

### Factors associated with sex-differences in clinical outcome

As shown in Table 5, the OR reduced by 45%, 50%, 58%, 34%, 2% and 26% for cardiovascular mortality after sequential cumulative adjustment for age, socioeconomic status, duration since symptom onset to emergency department and the approach to emergency department, management at emergency department, medical management in inpatient department, and procedural characteristics, respectively. The OR reduced by 18%, 22%, 17%, 31%, 3% and 11% for ADHF after sequential cumulative adjustment for covariates of these six domains. These findings indicated that socioeconomic status, and duration since symptom onset to emergency department and the approach to emergency department were the two main factors contributing to the sex-difference in cardiovascular mortality, while socioeconomic status and management in the emergency department were the two main factors contributing to the sex-difference in ADHF.

**Table 5:** Factors associated with sex-difference in clinical outcome

Clinical outcome	CV mortality	Non-CV mortality	ADHF
*Odds ratio and 95% CI*			
Unadjusted	2.57 (1.09–6.27)	1.60 (0.17–15.5)	1.65 (1.02–2.66)
Model 1	2.12 (1.04–4.39)	1.21 (0.33–8.96)	1.47 (0.95–2.08)
Model 2	1.62 (0.90–2.23)	1.08 (0.41–4.29)	1.25 (0.86–1.60)
Model 3	1.04 (0.78–1.44)	1.01 (0.43–2.63)	1.08 (0.73–1.24)
Model 4	0.70 (0.62–1.20)	0.79 (0.36–1.82)	0.77 (0.68–1.13)
Model 5	0.68 (0.57–1.13)	0.74 (0.35–1.76)	0.74 (0.64–1.08)
Model 6	0.42 (0.37–1.02)	0.65 (0.40–1.61)	0.63 (0.55–1.01)

Model 1: age; Model 2: model 1 plus educational attainment, and health insurance; Model 3: model 2 plus symptom onset to emergency department, and using emergency medical service to emergency department; Model 4: model 3 plus ticagrelor loading, duration since arrived at emergency department to first ECG test, duration since arrival at emergency department to first Hs-cTNT test, and duration since arrival at emergency department to Cardiologist consultation; Model 5: model 4 plus ticagrelor, statins, betablocker, renin-angiotensin-system inhibitor, and antidiabetics; Model 6: model 5 plus ST-segment elevation myocardial infarction, duration since symptom onset to undergo PCI, number of coronary arteries ≥ 70% stenosis, lesion length and location, post-PCI TIMI flow, and glycoprotein IIb/IIIa inhibitor

CV, cardiovascular; ADHF, acute decompensated heart failure

## Discussion

To the best of our knowledge, the current study should be the first few studies to evaluate the sex-differences in the management at emergency department and in-hospital clinical outcome in Chinese ACS patients. There are three main findings of the current study. First, compared to men, women received less effective and efficient treatment at the emergency department, which in turn might contribute to a worse prognosis in women. Second, the higher in-hospital rate of cardiovascular mortality and ADHF in women was disappeared after adjusting for covariates. Third, sequential cumulative regression analyses indicated that socioeconomic status, duration since symptom onset to emergency department and the approach to the emergency department, and management at emergency department were the three main factors contributing to the worse prognosis in women.

Acute coronary syndrome causes substantial health and economic loss in China and worldwide [3–5]. It is well-recognized that immediate and effective management are essential to improve clinical outcome for ACS patients. With advancement in therapy, the prognosis of ACS patient is improved in the last two decades. Nevertheless, there were substantial sex-differences in the improvement. For example, Raphael et al. reported that among individuals undergoing PCI, women had a higher mortality rate than men [10]. Using data from 21 randomized PCI trials, Kosmidou et al. reported that female sex was an independent predictor of major adverse cardiovascular events [18]. One recent study from Chinese ACS populations undergoing PCI indicated that women had a higher in-hospital mortality rate than men [7]. Using data from China Patient-Centered Evaluative Assessment of Cardiac Events (PEACE), Dreyer et al. reported that women in China had higher crude rates of all-cause and cardiovascular mortality even after adjusting for multiple covariates [9]. One recent meta-analysis showed that P2Y12 inhibitor monotherapy lowered the risk of the primary ischemic endpoint in women but not in men [19], suggesting the sex-specific effect of antiplatelet therapy in patients with revascularization. Results of the current study showed that women had a higher crude risk of cardiovascular mortality and ADHF than men. However, after adjusting for other covariates, there was no sex-differences in clinical outcomes, suggesting that female sex per se was not an independent risk factor for worse prognosis.

Extending from prior reports, the current study showed that women received less optimal treatment at the emergency department. In specific, compared to men, it took a longer duration for women to have electrocardiogram and Hs-cTnT test, which in turn might lead to delayed cardiologist consultation and a longer waiting duration to undergo PCI. The reason to explain the delay to undergo electrocardiogram and Hs-cTnT test in women might be partly due to their unspecific symptoms. Indeed, one recent meta-analysis has shown that compared to men, women with ACS were less likely to present with chest pain and diaphoresis, while they were more likely to present with pain between the shoulder blades or with gastrointestinal symptom [20]. Continued education and flowchart highlighting the unspecific symptoms of ACS women at the emergency department may help avoid the delay of performing electrocardiogram and Hs-cTnT test for ACS women. In addition, women were less likely to receive potent antiplatelet drug (ticagrelor) loading at the emergency department. These differences together might contribute to the worse prognosis in women. Indeed, immediate cardiologist consultation and potent antiplatelet drug loading are two essential factors associated with the prognosis for ACS patients [5, 21]. Interestingly and importantly, one recent meta-analysis has evaluated whether there was difference in ischemic events between P2Y12 inhibitor monotherapy or dual antiplatelet therapy after coronary revascularization [19]. The results suggested that monotherapy was associated with lower risk of ischemic events in women but not in men, suggesting that monotherapy might be sufficient for women but not for men. Biological differences in response to antiplatelet therapy by sex might partly explain these observations. However, due to the subgroup analysis, these findings can only be used for hypothesis generation and should be confirmed in clinical trial.

Factors from different domains have been ascribed to explain the observed sex-differences in clinical outcome. For example, Ho et al. reported that the sex-differences in in-hospital mortality were mainly due to worse clinical profiles and fewer evidence-based treatments for ACS women [7]. Cenko et al. reported that delayed hospitalization was independently associated with excess mortality in women [22]. The higher mortality rate in women undergoing PCI were due to their older age and greater comorbid burden [10]. Batchelor et al. reported that women experienced a higher risk of recurrent ischemic events after PCI treatment, which were primarily due to their lower socioeconomic status [23]. Leveraging data from the Longitudinal Assessment of Treatment Patterns and Events after Acute Coronary Syndrome (TRANSLATE-ACS) cohort, Hess et al. reported that differences in demographic, clinical, and treatment profiles explained the higher rate of major adverse cardiac event in women [24]. We observed that socioeconomic status, and duration since symptom onset to emergency department and the approach to emergency department were the two main factors associated with sex-differences in cardiovascular mortality. In specific, women had a lower literacy and were less likely to have health insurance, which might be associated with the delay to emergency department and lower use of EMS. Indeed, low socioeconomic status is an independent risk factor of poor prognosis, and the reasons to explain these observations which included low health literacy, poor health insurance, nonadherence to medication use, lack of access to healthcare, insufficient family support among others [25, 26]. While for ADHF, socioeconomic status and management at emergency department were the two main factors explaining the observed sex-differences. Indeed, the delay for first electrocardiogram and Hs-cTNT test and cardiologist consultation might be associated with delay of undergoing PCI, which in turn caused more severe myocardial injury. Women had a higher serum level of Hs-cTNT, suggesting a greater myocardial necrosis and a higher risk of developing HF [27]. Higher level of NT-proBNP level, a parameter of cardiac stress, portends an increased risk of HF [28, 29]. Taken together, prior and the current studies suggested that the mechanisms underlying sex-difference in clinical outcome among ACS patients undergoing PCI are complex and multifactorial. Improvement of management at the emergency department for women may be beneficial to reduce the sex-differences in clinical outcome.

## Limitations

There are some limitations of the current study. First, this was a retrospective study and findings from the current study should not be drawn for any causal relationship. Second, although we had extensively adjusted for covariates, unmeasured and unknown covariates (e.g. estrogen and menopausal status) might influence the relationship between sex and clinical outcomes. Third, this was a single center study from Chinese populations. Large sample size study from multiple center and different population groups are needed to corroborate the current findings. Fourth, it is unknown whether the sex-differences in in-hospital clinical outcome would extend to a long-term follow-up. Further study with long-term follow-up is warranted.

## Conclusion

In conclusion, findings of the current study suggest that among ACS patients undergoing PCI, there was no sex-difference in in-hospital clinical outcome after adjusting for covariates. Future studies are needed to evaluate whether improving management at the emergency department can improve clinical outcomes in women and men with ACS.

## Data Availability

The datasets used and/or analysed during the current study available from the corresponding author on reasonable request.
